# Topography of UV-Melanized Thalli of *Lobaria pulmonaria* (L.) Hoffm

**DOI:** 10.3390/plants12142627

**Published:** 2023-07-12

**Authors:** Amina G. Daminova, Anna E. Rassabina, Venera R. Khabibrakhmanova, Richard P. Beckett, Farida V. Minibayeva

**Affiliations:** 1 Kazan Institute of Biochemistry and Biophysics, FRC Kazan Scientific Center of RAS, 2/31, Lobachevsky Str., Kazan 420111, Russia; daminova.ag@gmail.com (A.G.D.); aerassabina@yandex.ru (A.E.R.); venerakhabirakhmanova@gmail.com (V.R.K.); 2 School of Life Sciences, University of KwaZulu-Natal, PBag X01, Scottsville 3209, South Africa; rpbeckett@gmail.com

**Keywords:** lichen, UV-induced melanization, melanin, topography, hydrolytic enzymes, microscopy

## Abstract

Lichens are unique extremophilic organisms due to their phenomenal resistance to adverse environmental factors, including ultraviolet (UV) irradiation. Melanization plays a special role in the protection of lichens from UV-B stress. In the present study, we analyzed the binding of melanins with the components of cell walls of the mycobiont of the upper cortex in the melanized lichen thalli *Lobaria pulmonaria*. Using scanning electron and atomic force microscopy, the morphological and nanomechanical characteristics of the melanized layer of mycobiont cells were visualized. Melanization of lichen thalli led to the smoothing of the surface relief and thickening of mycobiont cell walls, as well as the reduction in adhesion properties of the lichen thallus. Treatment of thalli with hydrolytic enzymes, especially chitinase and lichenase, enhanced the yield of melanin from melanized thalli and promoted the release of carbohydrates, while treatment with pectinase increased the release of carbohydrates and phenols. Our results suggest that melanin can firmly bind with hyphal cell wall carbohydrates, particularly chitin and 1,4-β-glucans, strengthening the melanized upper cortex of lichen thalli, and thereby it can contribute to lichen survival under UV stress.

## 1. Introduction

Lichens are supraorganismal symbiotic systems, comprising a thallus formed by two major partners, a fungus (the mycobiont) and an alga and/or a cyanobacterium (the photobiont) [1]. These two organisms are closely linked morphologically, physiologically, and biochemically [2,3]. Lichens may cover up to 100% of the ground at the sites where vascular plants are at their physiological limits [4]. Lichens are classified as extremophilic organisms due to their phenomenal resistance to adverse environmental factors, including ultraviolet (UV) stress [5]. Among the various defense mechanisms, the synthesis of secondary metabolites, including melanin pigments, plays a special role [6]. Melanization of the cortical layer of the lichen thallus prevents the damage of intracellular components during the exposure of lichen thallus to UV irradiation and high-intensity light. Melanins are dark pigments produced through the oxidation and polymerization of phenolic or indolic precursors [7,8]. The application of a combination of transmission and scanning electron microscopy methods has shown that melanins have a granular structure and occur as dark granules of different sizes [9,10]. The microstructure of melanins has been studied in several pathogenic fungi [11,12] and human melanosomes [13,14]. In fungi, melanins can be either secreted into the external environment or accumulate in the fungal cell wall [15]. High-resolution solid-state NMR has shown that, in *Cryptococcus neoformans*, melanins are probably covalently bound to cell wall chitin [16]. A close association between melanin and chitin has also been reported for *Aspergillus nidulans* [17], *Exophilia dermatitides* [18], and *Candida albicans* [19]. Fungi with mutated genes that are involved in the biosynthesis of the cell wall chitin or chitosan display a “leaky melanin” phenotype or possess enhanced pigment deposition [19]. The incorporation of melanin into the fungal cell wall decreases the pore size, reducing the conductivity of the wall, e.g., to water [20,21]. In our earlier study on UV-induced melanization of the lichen *Lobaria pulmonaria*, we visualized stages in the formation of melanin-like granules, including the formation of melanin vesicles, their transport, aggregation, and deposition of granules in cell walls of hyphae of the upper cortex [8,10]. However, information about the ability of melanin to bind with cell wall components of lichen mycobionts is scarce. This is likely a result of the complexity of the anatomical structure of the lichen thallus, the interactions between the fungal and photosynthetic symbionts, and the diversity of metabolite biosynthesis pathways that occur in lichens. Furthermore, lichen melanins can form complexes with metals and the elements of lichen cell walls, making them difficult to study [6,22]. Previous studies on melanin complexes isolated from two lichen species used infrared (IR) spectroscopy to reveal the presence of aromatic and aliphatic functional groups [23]. It is known that treatment of the cell wall with hydrolytic enzymes is a relatively mild but effective method of breaking down cell walls with high specificity [24]. For example, treatment of the fungi *Inonotus hispidus* with complex hydrolyzing enzymes resulted in an increased yield of extracted melanin [25].

Understanding the structure and properties of melanin associations in the cell walls is important for understanding how melanin strengthens lichen thalli and facilitates tolerance of lichen to UV stress. The aim of the present study was to visualize the topography of UV-melanized thalli and to analyze the binding of melanins with the components of cell walls in *Lobaria pulmonaria* (L.) Hoffm., a large leafy epiphytic lichen, which predominantly grows at a height of 1–2 m on wet bark of the lower part of the trunks of deciduous and coniferous trees. The species has an extensive range, covering Europe, Asia, Africa, North America and Australia. The thallus of the lichen *L. pulmonaria* represents an association of mycobiont hyphae (ascomycetes) with photobiont cells (green algae *Symbiochloris reticulata* and cyanobacteria *Nostoc*) [26,27]. Cyanobacteria of the genus *Nostoc* are included in special structures termed cephalodia and carry out biological fixation of atmospheric nitrogen. The upper cortex of sun-exposed thalli of *L. pulmonaria* can be dark brown, while shade-adapted thalli are normally bright green. The lower cortex is beige or light brown. Field experiments have shown that exposing shade-adapted *L. pulmonaria* to normal solar radiation induces L-DOPA melanin synthesis [28,29].

To study the morphological and nanomechanical characteristics of the melanized layer of mycobiont cells, scanning electron microscopy (SEM) and atomic force microscopy (AFM) were applied. SEM reproduces the lateral dimensions of thalli, while AFM can provide direct information on the relief or “height” and adhesion of the surface. We hypothesized that the changes in the relief and adhesion of the surface of thalli that have melanized following exposure to UV-B result from the association of melanins with polysaccharides, especially chitin and β-glucans, and proteins in the cell walls. To test this, hydrolyzing enzymes were applied to melanized lichen thalli. The ability of the enzymes to break down the bonds was assessed by measuring the release of carbohydrates, proteins, and phenols, and the yield of melanin. Changes caused in the ultrastructure of the cells in the upper cortex following enzyme treatments were visualized using transmission electron microscopy (TEM).

## 2. Results

### 2.1. Morphology

**Figure 1:**
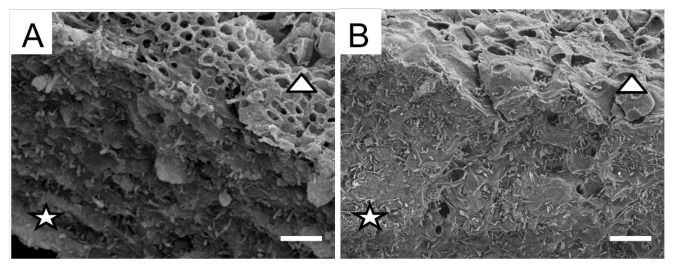
SEM images of the anticlinal (stars) and periclinal cell walls (triangles) of cross sections of pale (**A**) and melanized (**B**) thalli of *L. pulmonaria*. Scale bar corresponds to 10 µm.

Analysis of the surface topography of cross sections from the upper cortex of pale and melanized *L. pulmonaria* thalli by SEM revealed that the relief of the anticlinal cell walls of the pale thallus was heterogeneous and rough and characterized by a large number of depressions and bulges (Figure 1A). The anticlinal cell walls of melanized thallus were smoother, with fewer differences in height. Cell walls of the melanized thallus were visibly thicker than those in the pale thallus (Figure 1A,B).

**Figure 2:**
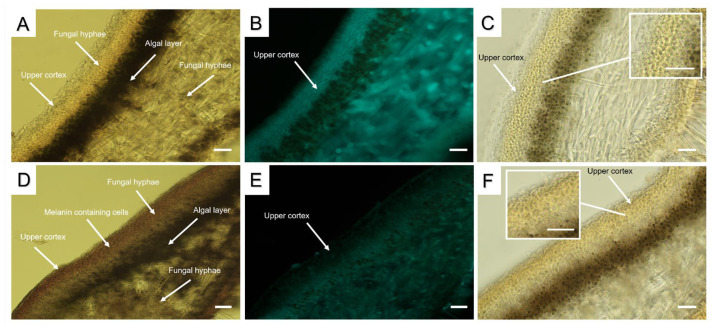
Cross sections of pale (**A**–**C**) and melanized (**D**–**F**) thalli of *L. pulmonaria*: nonstained cross sections (**A**,**D**); Calcofluor staining (**B**,**E**); Lugol staining (**C**,**F**). Scale bar corresponds to 25 µm.

By qualitative reactions using Calcofluor staining and Lugol staining, we visualized the presence of carbohydrates, including chitin, in the cell walls of cortical cells in the cross sections of pale and melanized *L. pulmonaria* thalli. Interestingly, while in the pale thalli Calcofluor staining was very bright (Figure 2B), fluorescence in the melanized thallus was much weaker (Figure 2E). Lugol staining of chitin demonstrated the presence of the small light purple grains in the upper cortex of pale thalli (Figure 2C), whereas pigmentation of the upper cortex of melanized thalli was darker (Figure 2F).

**Figure 3:**
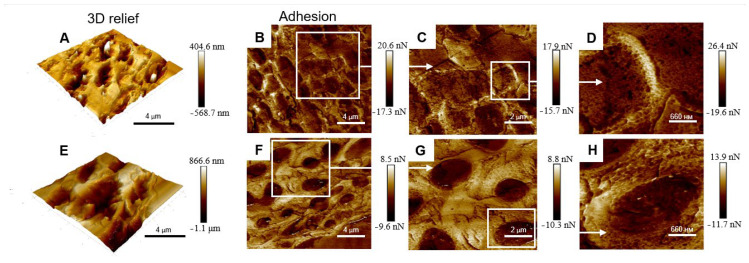
3D relief (**A**), adhesion (**B**–**D**) of the surface of cross sections of nonmelanized (**A**–**D**) and 3D relief (**E**), adhesion (**F**–**H**) of the surface of cross sections of melanized (**E**–**H**) *L. pulmonaria* thalli.

To study the topographical features of the upper cortex, we analyzed the 3D relief and adhesion of the surface of the cross sections of *Lobaria* pale and melanized thalli using AFM (Figure 3). Melanized samples displayed approximately twice the variation in height variations, while adhesion values were reduced by 0.6 compared to those of the pale samples. An increase in height variation of the upper cortex of melanized thalli measured by AFM (vertical dimension, Figure 3A,E) corresponded to an increase in their SEM widths (lateral dimension, Figure 1A,B). Visualized differences in the adhesion in pale (Figure 3B–D) and melanized thalli (Figure 3F–H) corresponded to the differences in the morphology of the cell walls in pale and melanized thalli, as shown in Figure 1A,B. Thus, SEM and AFM imaging gave complementary information.

### 2.2. The Effect of Enzyme Treatment on the Release of Components

**Figure 4:**
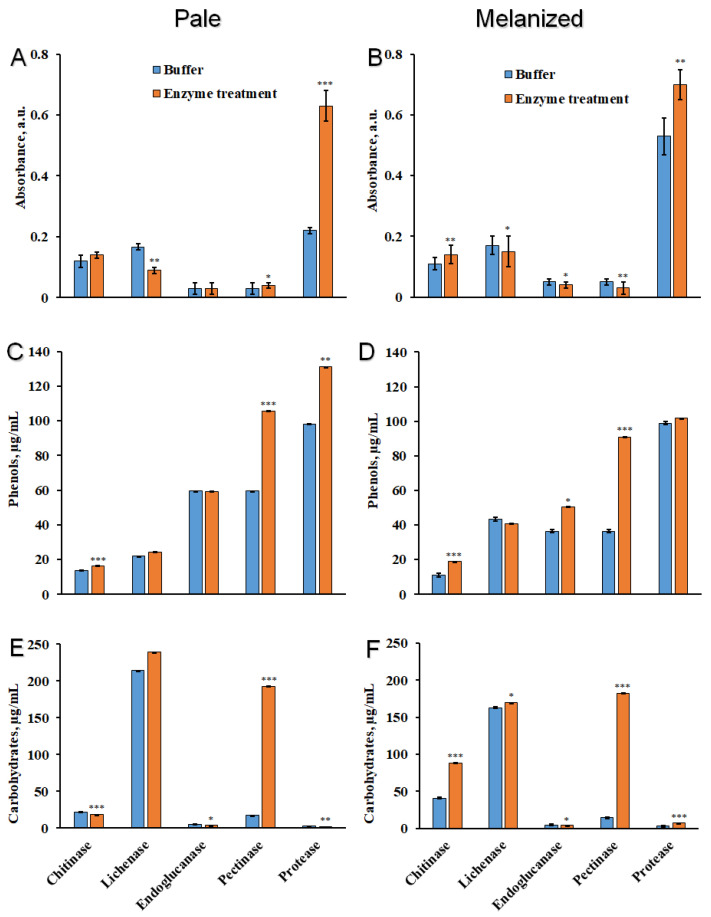
Absorbance of the incubation solutions at wavelength 490 nm (λ_490_, (**A**,**B**)), the content of total phenolic compounds (**C**,**D**) and total carbohydrates (**E**,**F**) of pale (**A**,**C**,**E**) and melanized (**B**,**D**,**F**) thalli of lichen *L. pulmonaria* treated with buffer solutions with and without enzymes (*n* = 3). Statistically significant differences are indicated as *p* ≤ 0.05 (*), ≤0.01 (**), ≤0.001 (***).

To characterize the binding of melanins with the components of cell walls in *L. pulmonaria*, discs of pale and melanized lichen thalli were incubated in the solutions with enzymes that hydrolyze the bonds between melanin and carbohydrates, proteins, and phenols. The list of enzymes, their specific activity, corresponding buffers and concentrations are presented in Table 1. After treatment of pale and melanized thalli with enzymes, the absorbance of the incubation solutions was analyzed at a wavelength of 490 nm (Figure 4A,B), which corresponds to the absorbance of melanins [22] and some other phenolic compounds such as quinones [30]. After treatment of thalli with chitinase, lichenase, endoglucanase, and pectinase, the absorbance of incubation solutions was low, and there was no difference between pale and melanized thalli. However, treatment of the thalli with protease caused a significant increase in absorbance, especially of the incubation solution of melanized lichen (Figure 4B). Interestingly, incubating both types of lichen thalli in the buffer without protease also increased the absorbance. Following incubation in solutions containing buffer only, the absorbance of melanized thalli was more than double that of pale thalli (Figure 4A,B).

Treatment of the pale thalli with pectinase and protease increased the release of phenolic compounds into incubation solutions (Figure 4C). The release of phenolic compounds from melanized thalli was highly stimulated by pectinase (Figure 4D). The significant release of phenolic compounds from melanized thalli was observed following incubation of discs in the solutions with and without protease (Figure 4D). After the incubation of lichen thalli in the solutions containing chitinase, lichenase, and endogluconase, the release of phenolic substances to the incubation solutions was low and similar to their release into solutions containing only buffers.

Treatment of pale thalli with buffers with or without chitinase releases small amounts of carbohydrates (Figure 4E), while the release of total carbohydrates from melanized lichen thalli doubled after treatment with chitinase (Figure 4F). The treatment of pale and melanized lichen thalli with buffers with and without lichenase greatly increased the release of carbohydrates (Figure 4E,F). The release of carbohydrates was not observed after treatment of pale and melanized thalli with endogluconase and protease, while the incubation with pectinase resulted in the significant release of carbohydrates (Figure 4E,F).

**Figure 5:**
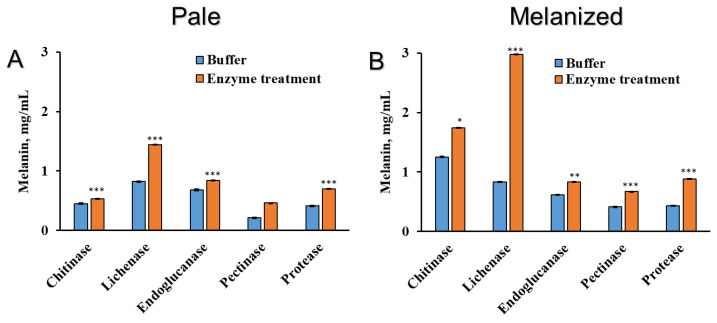
Content of melanin (mg dry melanin/mL alkaline solution) in the pale (**A**) and melanized (**B**) thalli of the lichen *L. pulmonaria* following enzyme treatments. Statistically significant differences are indicated as *p* ≤ 0.05 (*), ≤0.01 (**), ≤0.001 (***).

Measuring the melanin after enzyme treatments showed that the general contents were lower in pale than melanized thalli (Figure 5A,B). After treatment of melanized thalli with chitinase, and especially lichenase, the yield of melanin was much higher compared to that of solutions that contained only buffer (Figure 5B). Endoglucanase marginally increased the amount of melanin that could be extracted, while pectinase and protease faciliated the increase of more melanins from both pale and melanized thalli.

### 2.3. The Ultrastructural Characteristics of the Cross Sections of Melanized Lichen Thalli

**Figure 6:**
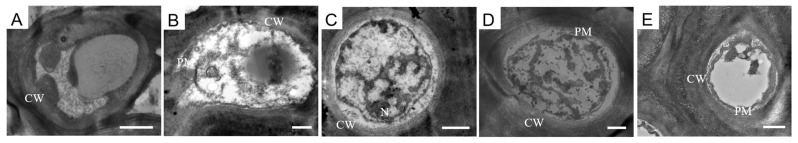
Transmission electron microscopy images of cross sections of melanized *L. pulmonaria* thalli: without enzyme treatment (**A**), after treatment with chitinase (**B**), endoglucanase (**C**), pectinase (**D**), and protease (**E**). Cell wall (CW), plasma membrane (PM), nucleus (N). Scale bar corresponds to 0.5 µm.

After treatment of melanized *L. pulmonaria* thalli with chitinase, endoglucanase, pectinase, and protease, we analyzed the ultrastructural properties of cross sections of these thalli by TEM (Figure 6). In melanized thallus, the fungal hyphae had thick cell walls with a clearly visualized melanic layer and an intact plasma membrane (Figure 6A).

Treatment of the thalli with enzymes caused significant changes in the cellular ultrastructure. In particular, layering and loosening of the cell walls (Figure 6C–E), and decondensation of interhyphal space (Figure 6C,D), were observed. Furthermore, the integrity of the plasma membrane was disrupted (Figure 6B–E), and it became detached from the cell wall of the hyphae; significant plasmolysis of the cellular contents was visualized (Figure 6B–E).

## 3. Discussion

The ability of lichens to synthesize darkly pigmented melanins contributes to their survival in hostile environments, including UV stress and desiccation [31,32]. In the present study, by applying advanced imaging techniques such as AFM, we discovered that UV-B irradiation causes changes in the topography of thalli of the lichen *L. pulmonaria*. Following UV irradiation, *L. pulmonaria* becomes melanized, and compared to pale thalli, anticlinal and periclinal cell walls of melanized upper cortex become visibly thicker and display a smoother relief (Figure 1). In our previous paper, we showed that UV induces the formation of melanin-like granules in the hyphae in the melanized upper cortex of lichen thallus [10]. We suggest that the changes in morphology of thallus surface during melanization result from the formation of melanin complex polymers in the cell walls of the fungal hyphae in the upper cortex.

Studying the structure of melanins is challenging, because they are complex polymers, insoluble, amorphous, and heterogeneous, comprising a mixture of proteins, carbohydrates, including chitin/chitosan, and lipid moieties [33,34]. These compounds appear to serve as scaffolds for melanin synthesis [16,35]. Wall-bound electron-dense melanin granules may occur in the outer or inner part of the cell walls of fungal hyphae [36]. In melanized thalli of *Lobaria*, the upper cortex is visibly much darker than that of pale thalli (Figure 2A,D). To visualize the association of melanin with other components of the hyphal cell walls, qualitative stains such as Calcofluor for carbohydrates and Lugol for chitin were used. Compared to melanized thalli, pale thalli displayed more intense Calcofluor staining in the upper cortex (Figure 2B,E). It seems likely that bonding between melanins and polysaccharides impedes the normal interaction of the stain with its target molecules. In cross sections of the melanized thalli of *L. pulmonaria*, staining with Lugol results in a dark purple stippling (Figure 2C,F), indicating the presence of chitin in the upper cortex. Chitin, a long-chain polymer of N-acetylglucosamine, provides mechanical strength to fungal cell walls and is therefore an important structural component of the cell wall [37,38]. Several studies suggest that chitin is a primary effector for melanin polymer deposition within the fungal cell wall [39,40,41,42].

Investigating the mechanical properties of tissues at the cellular level by AFM can greatly assist in understanding the processes of growth and morphogenesis [43]. For example, in maize primary roots, stiffness and elasticity of the stele vascular parenchyma periclinal cell walls are correlated with cell wall mechanical properties [44]. There have been several studies on the conformation of melanins using ASM, for example, the ultrastructural characteristics of eumelanin from *Sepia officinalis* [45,46]. In the present study, imaging the topography and mechanical properties of melanins in the thallus of *L. pulmonaria* by ASM demonstrates that UV-B-induced melanization changes the physical parameters of the upper cortex. The height of the surface of melanized samples increases, while adhesion is reduced compared to that in the pale thalli (Figure 3). Adhesion is an integral parameter comprising both the adherence of the thalli to the AFM probe and the contribution of the interfacial water film. Yet, we see that the adhesion force is different for these two types of thalli. This observation indirectly supports the difference in the topography of two thalli at the nanoscale. In the green alga *Enteromorpha linza* (L.), AFM was used to show that glycoprotein is a natural adhesive and provides firm anchorage to the substratum [47]. It is likely that the changes in the height and adhesion of the surface of melanized samples result from the association of melanins with polysaccharides and proteins in the cell walls.

To study the nature of the link between melanins and cell wall components, we treated pale and melanized lichen thalli with chitinase, lichenase, endoglucanase, pectinase, and protease. These enzymes can hydrolyze the glucosidic bonds of structural carbohydrates of the cell wall and also proteins (Table 1). Treatment with pectinase causes a significant release of carbohydrates and phenols, although pectin substances are not common in lichens (Figure 4). However, some lichens, e.g., *Evernia prunastri* (L.) Ach. [48] and *Peltigera canina* (L.) Willd. [49], contain pectinases, specifically polygalacturonase, which may indicate that some pectins are present. *Lobaria pulmonaria* may contain pectin or pectin-like polysaccharides that can be target molecules for pectinase. Interestingly, incubation in buffer solutions with a pH of 7.4 can induce the release of phenolic compounds from melanized lichen thalli, possibly indicating that they are soluble in alkaline solutions [50,51].

Lichen polysaccharides are mainly linear or weakly substituted *α*- or β-glucans [52]. Therefore, it is not surprising that treatment of melanized lichen thalli with chitinase and lichenase increases the release of carbohydrates (Figure 4E,F). Furthermore, the yield of melanin from melanized thalli was much higher following treatment with chitinase, and even more so lichenase, which hydrolyzes 1,4-β-glucosidic bonds (Figure 5). By contrast, endoglucanase, which hydrolyzes 1,3-β-glucosidic bonds, did not increase in the yield of melanin extracted (Figure 5). Therefore, these results suggest the presence of chitin and 1,4-β-glucans in the cell wall of mycobiont. Protease only had a small effect on the release of melanin. It seems likely that melanin is strongly associated with the structural components of the cell wall that are targeted by hydrolytic enzymes. Hydrolytic enzymes also cause changes in the ultrastructure of hyphal cells of the upper cortex, such as layering and loosening of the cell walls, decondensation of interhyphal space, and significant plasmolysis of the cellular content (Figure 6). Taken together, the observed ultrastructural changes and the effects of hydrolytic enzymes on the release of compounds from the thalli strongly suggest that bonds exist between melanin and structural polysaccharides in the cell wall. Future research is required to determine the structure, morphology, and physicochemical properties of isolated associations of lichen melanins with cell wall polysaccharides.

## 4. Materials and Methods

### 4.1. Sample Preparation

Pale and melanized thalli of *L. pulmonaria* (L.) Hoffm. were collected from the bark of poplar trees growing in the outskirts of Syktyvkar, Komi Republic, Russia (latitude 61′34’ N, longitude 50′33’ E). Lichen material was cleaned, slowly dried at room temperature, and then stored at −20 °C. Samples were chosen from the medial zone of the thallus, away from the margins and the basal thalline zone attached to the substratum. Before experiments, 1 cm disks were cut, their dry mass determined, and then placed on wet filter paper and hydrated in a climate chamber at +15 °C and 12/12 h (light/dark). The thalli were irradiated by UV-B (λ = 280–315 nm) fluorescent erythema lamps (3 W m^−2^) for 80 min daily for 14 d. During this time, the controls were taken out of the climate chamber and kept under regular laboratory conditions without UV irradiation.

### 4.2. Scanning Electron Microscopy

To image melanin of *L. pulmonaria* by scanning and transmission electron microscopy, thalli were embedded in 3% agarose blocks and cut using a vibratome (Leica VT 1000S, Wetzlar, Germany), resulting in 50 µm cross sections from the upper cortex. The cross sections of pale and melanized lichen thalli were subsequently fixed in 2.5% glutaraldehyde in 0.1 M Na phosphate buffer, pH 7.4, and 1% osmium tetroxide, and further dehydrated as described by Daminova et al. (2022) [10]. Sections were then sputter-coated with gold using the Q150T ES Coater (Quorum Technologies, Lewes, UK), and anticlinal and periclinal cell walls were viewed using a high-resolution scanning electron microscope (Merlin, Carl Zeiss, Oberkochen, Germany) at a voltage of 5 kV.

### 4.3. Atomic Force Microscopy

Disks of lichen thalli were fixed according to standard protocols [53]. Briefly, the samples were fixed in glutaraldehyde (Sigma, St. Louis, MO, USA), post-fixed in osmium tetroxide (Sigma, USA), dehydrated in a graded aqueous ethanol series and acetone, embedded in LR White resin (Medium Grade Acrylic Resin; Ted Pella, Redding, CA, USA), and polymerized at 60 °C for 24 h. Samples were carefully inserted in a vertical orientation in beam capsules, which were then filled with LR White resin before polymerization [54]. The top of the sample blocks was leveled using a diamond knife (Electron Microscopy Sciences, Hatfield, PA, USA). The upper cortex was visualized using the Bruker Dimension FastScan microscope (Bruker, Billerica, MA, USA) in PeakForceQNM (quantitative nanomechanical mapping) mode [55]. To obtain high-quality images of the topography and nanomechanical characteristics of the sections, the standard silicon cantilevers ScanAsyst-Air (Bruker, Billerica, MA, USA) with curvature 2 nm and stiffness 0.4 N m^−1^ were used. Images were acquired at a resolution of 512 lines per scan [56].

### 4.4. Light Microscopy

For light microscopy, pale and melanized lichen thalli were incubated in 2 M NaOH for 1 h at 100 °C, the pH adjusted to 7.0 with 5 M sulfuric acid, and then thalli were rinsed three times with distilled water. Cross sections were vibratome-cut, stained with Lugol solution and 10% sulfuric acid as described by Yajima et al. (2001) [57], and viewed with a bright-field epifluorescence microscope (Leica DM1000, Leica Biosystems, Wetzlar, Germany).

For fluorescence microscopy, cross sections of pale and melanized thalli were stained with 0.1% Calcofluor White (Sigma-Aldrich, St. Louis, MO, USA) for 15 min in the dark and visualized using an excitation wavelength of 380 nm and an emission wavelength of 475 nm using the epifluorescence microscope Leica DM1000. Images were taken by digital camera at a magnification of ×40.

### 4.5. Enzyme Treatments

Hydrated discs (on average c. 1.1 g) of *L. pulmonaria* thalli were vacuum-infiltrated in a 3 mL solution of the enzymes chitinase, lichenase, endoglucanase, pectinase, and protease (Table 1) three times for 1 min. After that, discs in solutions were shaken in the incubator (ES-20/60, BioSan, Riga, Latvia) at 170 rpm for 12–14 h in the dark at 37–40 °C for lichenase and at 25 °C for chitinase, endogluconase, pectinase, and protease. As a control, hydrated discs of lichen thalli were incubated in 3 mL of the corresponding buffer without enzyme.

### 4.6. Determination of the Content of Compounds Released into Incubation Solutions

After enzyme treatment, discs of lichen thalli were removed from the incubation solutions, rinsed with 5 mL of distilled water, and gently blotted with filter paper. Incubation solutions were centrifuged at 8000 rpm for 1.5 min using an Eppendorf MiniSpin (Eppendorf, Germany). The absorbance of the incubation solution was determined at 490 nm using the UV-1900 spectrophotometer (Shimadzu, Kyoto, Japan). The content of the total phenolic compounds was determined using the Folin–Ciocalteu reagent (Sigma-Aldrich, St. Louis, MO, USA) [58], calibrated with gallic acid (20–200 µg ml^−1^) (DiaM, Moscow, Russia). The content of nonreducing sugars was determined using the anthrone method [59], calibrated with glucose solutions (20–100 µg ml^−1^) (Reakhim, Moscow, Russia).

### 4.7. Content of Melanin in Lichen Thalli

Alkali-soluble metabolites were extracted from the thallus discs of *L. pulmonaria* after enzyme treatment using the protocol outlined by [51] with modifications. Initially, discs were homogenized with liquid N_2_ and then 2 M NaOH added in the ratio of 1:10. After alkaline extraction for 12 h at room temperature, the resulting homogenate was centrifuged at 8000 rpm for 10 min (Hermle, Gosheim, Germany). Melanin was precipitated from the alkaline extracts by adding HCl till pH 2 and incubating for 12 h at room temperature. After centrifugation, the resulting precipitate was oven-dried at 60 °C for 1 h and weighed. Melanin content was calculated in mg ml^−1^ of alkali extracts.

### 4.8. Transmission Electron Microscopy

The discs of melanized lichen thalli of *L. pulmonaria* after enzyme treatment were fixed according to the standard protocol (see above section AFM). After ultrathin sectioning of samples using an ultramicrotome (Leica, Wetzlar, Germany), cross sections were stained with 2% aqueous uranyl acetate (*w*/*v*) for 20 min and Reynolds’ lead citrate for 7 min [60]. Finally, the cross sections were examined using the Excellence transmission electron microscope (Hitachi HT 7700, Tokyo, Japan) at an accelerating voltage of 100 kV.

### 4.9. Statistical Analysis

Microscopical observations were performed using at least 6 representative samples, while all biochemical measurements were conducted in triplicate (*n* = 3). The values are expressed as mean ± standard deviation (SD). Statistical analyses were performed using the Student’s *t* test. The difference is statistically significant at *p* ≤ 0.05 (*), ≤0.01 (**), ≤0.001 (***).

## 5. Conclusions

The present study demonstrates that UV-B irradiation induces melanization and changes the topography of the lichen *L. pulmonaria* thalli. Accumulation of melanin results in a smoother relief with visibly thicker cell walls of the mycobiont upper cortex. Furthermore, melanization increases the height value and reduces the adhesion properties of the thallus. Treatment of thalli with hydrolytic enzymes, especially chitinase, lichenase, and pectinase, enhances the yield of melanin and the release of carbohydrates and phenols from melanized thalli, confirming the existence of bonds between melanins and cell wall components. Therefore, these results confirm our hypothesis that the association of melanins with polysaccharides, especially chitin and β-glucans, and proteins in the mycobiont cell walls can change the topography of the surface and properties of the melanized upper cortex. These findings shed light on the complex biomechanical mechanisms, by which melanins can strengthen lichen thalli and contribute to lichen survival under UV stress.

## Figures and Tables

**Table 1:** List of enzymes.

Enzyme	Specific Activity	Source	Buffer	Units mL^−1^
Chitinase ^1^	Hydrolysis of N-acetyl- β-D-glucosaminide (1→4)-β-linkages in chitin and chitodextrins	*Trichoderma viride*	50 mM sodium phosphate, pH 6.0	0.5
Lichenase ^2^	Hydrolysis of (1,4)-β-D-glucosidic linkages in β-D-glucans containing (1,3)- and (1,4)-bonds	*Bacillus subtilis*	10 mM sodium phosphate, pH 6.0	5
Endoglucanase ^2^	Hydrolysis of (1,3)-β-D-glucosidic linkages in (1,3)-β-D-glucans	*Trichoderma* sp.	100 mM sodium acetate, pH 4.5	0.5
Pectinase ^1^	Hydrolysis of pectin-containing substances	*Aspergillus aculeatus*	100 mM sodium acetate, pH 4.5	1
Protease ^1^	Hydrolysis of peptide bonds in proteins with conversion to shorter polypeptides and amino acids	*Bacillus licheniformis*	100 mM sodium phosphate, pH 7.4	1

^1^ Sigma-Aldrich, St. Louis, MO, USA. ^2^ Megazyme, Chicago, IL, USA.

## Data Availability

Not applicable.
